# Quality Improvement Project to Improve Screening for Tobacco Use in Adolescent Inpatients at a Children’s Hospital

**DOI:** 10.3390/children6030037

**Published:** 2019-02-28

**Authors:** Lauren A. Wagner, Adolfo L. Molina, Karisa Grizzle, Meghan E. Hofto, Lauren B. Nassetta, Mary M. Orr, Nichole Samuy, Erinn O. Schmit, Cassi Smola, Kathleen F. Harrington, Susan C. Walley

**Affiliations:** 1 Department of Pediatrics, University of Alabama at Birmingham and Children’s of Alabama, Birmingham, AL 35233, USA; amolina@peds.uab.edu (A.L.M.); kgrizzle@uabmc.edu (K.G.); mhofto@peds.uab.edu (M.E.H.); lnassetta@peds.uab.edu (L.B.N.); morr@peds.uab.edu (M.M.O.); nsamuy@peds.uab.edu (N.S.); eschmit@peds.uab.edu (E.O.S.); csmola@peds.uab.edu (C.S.); swalley@peds.uab.edu (S.C.W.); 2 Division of Pulmonary Medicine, Department of Medicine, University of Alabama at Birmingham, Birmingham, AL 35233, USA; kathyharrington@uabmc.edu

**Keywords:** tobacco, electronic cigarette, inpatient, adolescent, screening, hospital

## Abstract

Tobacco use begins in adolescence for the majority of smokers. The purpose of this study was to increase screening and reporting of tobacco use in hospitalized adolescents at a tertiary care children’s hospital. We completed a nursing focus group to understand challenges and completed four iterative Plan-Do-Study-Act cycles, which included: (1) in-person nursing education regarding tobacco use screening, (2) addition of an e-cigarette-specific screening question, (3) the creation and dissemination of an educational video for nursing, and (4) adding the video as a mandatory component of nursing orientation. Run charts of the percentage of patients screened who reported tobacco use were created. Absolute counts of tobacco products used were also captured. From January 2016 to September 2018, 12,999 patients ≥13 years of age were admitted to the hospital. At baseline, 90.1% of patients were screened and 4.8% reported tobacco use. While the absolute number of adolescents reporting e-cigarette use increased from zero patients per month at baseline to five, the percentage of patients screened and reporting tobacco use was unchanged; the majority of e-cigarette users reported use of other tobacco products. This study demonstrates that adding e-cigarettes to screening increases reporting and suggests systems level changes are needed to improve tobacco use reporting.

## 1. Introduction

Tobacco use and tobacco smoke exposure is the leading cause of preventable death and is associated with a multibillion dollar economic burden in the United States [1]. Over 90% of adult smokers begin using tobacco by age 19 years [1]. Nationally, over 24% of high schoolers report current use of tobacco products and almost 21% report current use of electronic cigarettes [2]. From 2017 to 2018, tobacco use dramatically increased in high schoolers by 38% for any type of tobacco and by 78% for e-cigarettes [2,3]. In Alabama, rates of youth and adult tobacco product use are higher than national averages. In 2015, 35.5% of Alabama high schoolers reported any type of tobacco use in the last 30 days compared to 31.4% nationally [4,5].

Children’s of Alabama (COA) is a free-standing tertiary care urban children’s hospital with approximately 400 adolescent patients admitted monthly. The United States Preventive Services Task Force (USPSTF) currently recommends tobacco smoking cessation and prevention services at the primary care level [6]. As adolescents have poor attendance at primary care visits, a hospitalization is an opportunity to address adolescent tobacco use. Many of these hospitalized adolescents have conditions which could be caused or exacerbated by tobacco use. 

With the ultimate goal of providing education, resources, and treatment for adolescent tobacco users, this project aimed to determine the rate of tobacco use screening of adolescents and the percentage of these youth who use tobacco. In addition, the tobacco use screen at our hospital did not initially include e-cigarettes, which is the most common form of tobacco used by youth [2]. Thus, this quality improvement (QI) project was initiated to address the identification of adolescent tobacco users at our institution. Specific aims of this QI project were (1) to increase screening for tobacco use to 95% of hospitalized patients 13 years and older, (2) to increase reporting of tobacco use to 20% in order to more accurately reflect reported tobacco use in Alabama, and (3) to increase reporting of electronic cigarette use to 10% in the 21 month intervention period in order to more accurately reflect known use numbers.

## 2. Materials and Methods

### 2.1. Context

**Figure A1:**
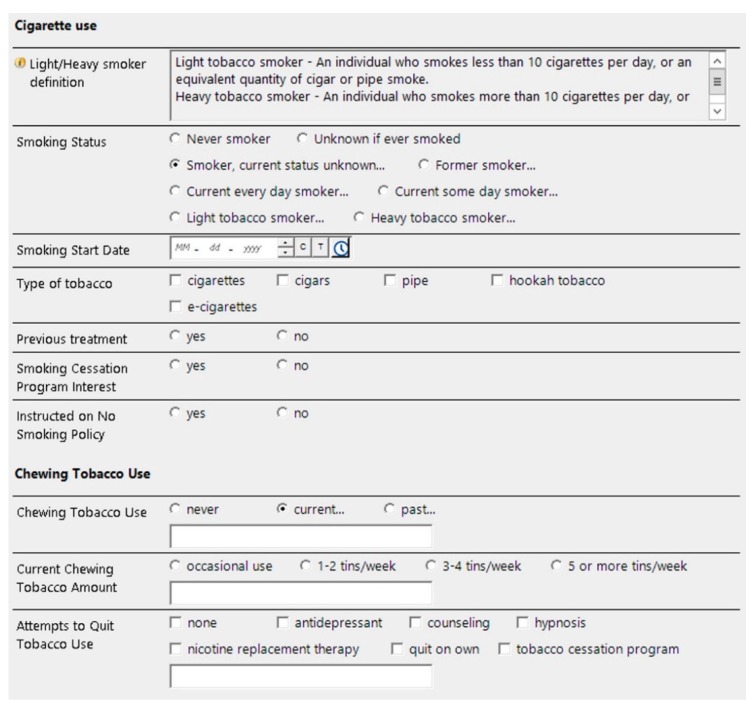
Tobacco use screening questions on admission Nursing Intake Form.

In 2012, the Joint Commission implemented Tobacco Cessation Performance measures that mandated comprehensive evidence-based tobacco dependence treatment for hospitalized adult patients [7]. While the Joint Commission’s tobacco measures were optional for accreditation (and did not apply to patients <18 years of age), they did highlight the opportunity to screen and treat hospitalized patients for tobacco use. COA had begun screening adolescents (≥13 years of age) by adding tobacco use screening questions to the admission Nursing Intake Form (as seen in Appendix A Figure A1), which is required to be completed by nursing within 24 hours of admission. 

In January 2017, workgroups with nursing leaders discussed optimization of adolescent tobacco use screening throughout the hospital. A front-line nursing focus group was conducted to discuss challenges regarding adolescent tobacco use screening. Nominal group technique was used for the focus group [8]. Nurses from many nursing units were included. The group identified and prioritized staff issues associated with tobacco use screening with adolescents, as seen in Table 1. 

### 2.2. Interventions

Utilizing themes from focus groups and implementing change with multiple cycles and tests of change are thought to produce more meaningful changes in attitudes and behavior over time [9]. As such, after getting background data and meeting with nursing leaders and a focus group with nurses, four Plan-Do-Study-Act iterative events occurred. First, in-person education to nursing groups on the importance of screening adolescents for tobacco use was performed. Second, e-cigarette use and hookah use were added to the tobacco use screening questions as separate options regarding type of tobacco. Third, an educational video geared towards nursing staff addressing the barriers identified in the nursing focus groups was created and disseminated. Fourth, the training video was made mandatory for inpatient nursing staff orientation.

### 2.3. Study of the Interventions

Data were obtained monthly and added to baseline data on the (individual vs. x-bar vs. R-chart) control charts created by QI macros 2018 (KnowWare International Inc., Denver, CO, USA) Excel extension. Data assessed were (1) percent of patients ≥13 years of age screened for tobacco use, and (2) percent of patients ≥13 years of age reporting tobacco use. We utilized the Institute for Healthcare Improvement control chart rules for assessing special vs. common cause variation [10]. In addition, we assessed the types of tobacco use reported. 

### 2.4. Measures

Baseline data were collected through electronic data abstraction to assess the monthly completion rate of tobacco use screening hospital-wide. Data collected include admission date, nursing unit, whether they were screened for tobacco use, results of the screen, and type of tobacco product used. Tobacco use screening data included never or ever smoker, quantity of use, and type of product used, including cigarettes, cigars, chewing tobacco, pipes, and ultimately e-cigarettes and hookah, as seen in Table 2. 

Patients unable to answer the questions were reported as NULL (not screened) in our data. Examples of medical conditions resulting in NULL responses were intubated patients, patients with altered mental status, or those with severe intellectual disability. It is also possible that some NULL responses were due to failure of form completion. Our goal for screening completion was 95% and is intentionally high, as it should only reflect the medical exceptions to mandatory screening within 24 hours of admission. 

### 2.5. Ethics

University of Alabama at Birmingham Institutional Review Board exemption for the nursing focus group study was obtained on 2 January 2017 with protocol number E161227003. No consent form was required as there was no personally identifying information collected. An information sheet was given to nurses explaining their participation in the study. As improving adolescent tobacco use screening was a QI study and not human subject research, it did not require IRB approval.

## 3. Results

**Figure 1:**
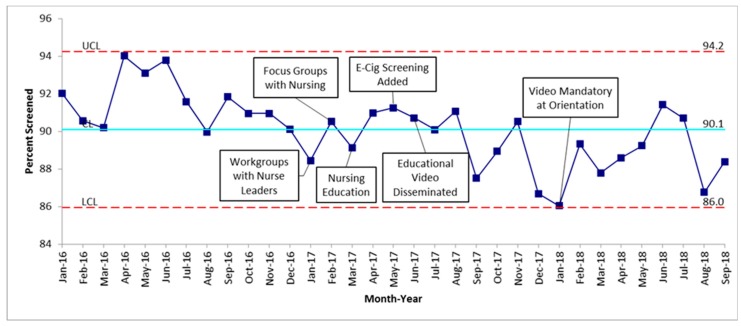
Inpatient adolescents screened for tobacco use. UCL—upper control limit is 3 sigma above the mean; LCL—lower control limit is 3 sigma below the mean. The blue line represents the mean.

**Figure 2:**
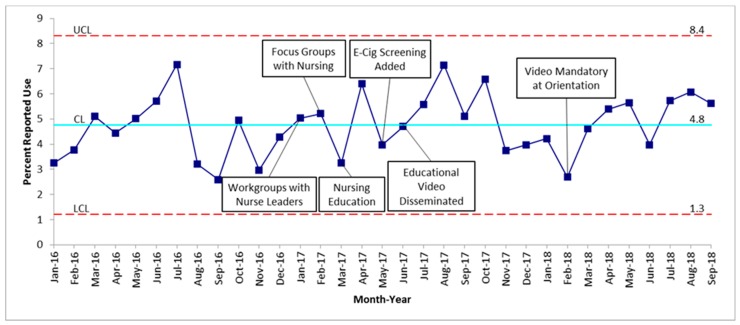
Inpatient adolescents reporting tobacco use. UCL—upper control limit is 3 sigma above the mean; LCL—lower control limit is 3 sigma below the mean. The blue line represents the mean.

Over the course of the 33 months (January 2016–September 2018) of data collection, 12,999 adolescent patients 13 years and older were admitted to medical/surgical and psychiatric units in our hospital. January 2016 through February 2017 established the baseline mean of patient screening at 90.1%, as seen in Figure 1. The baseline average of patients who reported tobacco use was 4.8%, as seen in Figure 2. The first nursing workgroup meetings and focus group took place in January and February 2017, with the intervention period consisting of 21 months from March 2017 through September 2018. The percent of adolescents screened and percent of adolescents reporting tobacco use was unchanged throughout the study period. 

**Figure 3:**
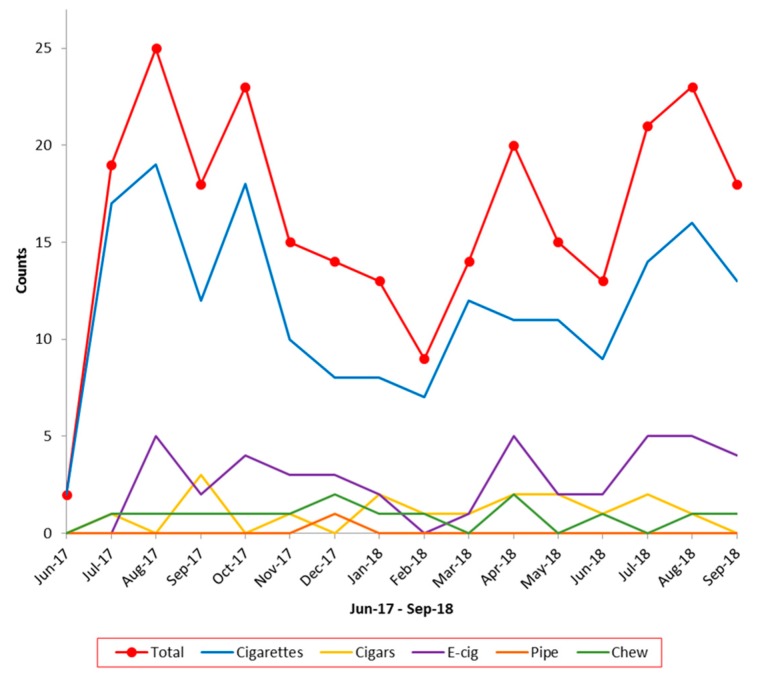
Absolute number of tobacco types used per month.

Absolute numbers of reported tobacco use during the 33 months among all patients screened were: cigarette—475 (4.05%), e-cigarette—46 (0.39%), chewing tobacco—43 (0.37%), cigar—26 (0.22%), pipe—2 (0.02%), and hookah—0 (0.0%). Some patients reported more than one type of tobacco used. With introduction of e-cigarettes to the tobacco use screen, the absolute number of patients reporting e-cigarette use increased from zero to five per month, as seen in Figure 3. However, this did not increase the overall percent of reported tobacco use. 

**Figure 4:**
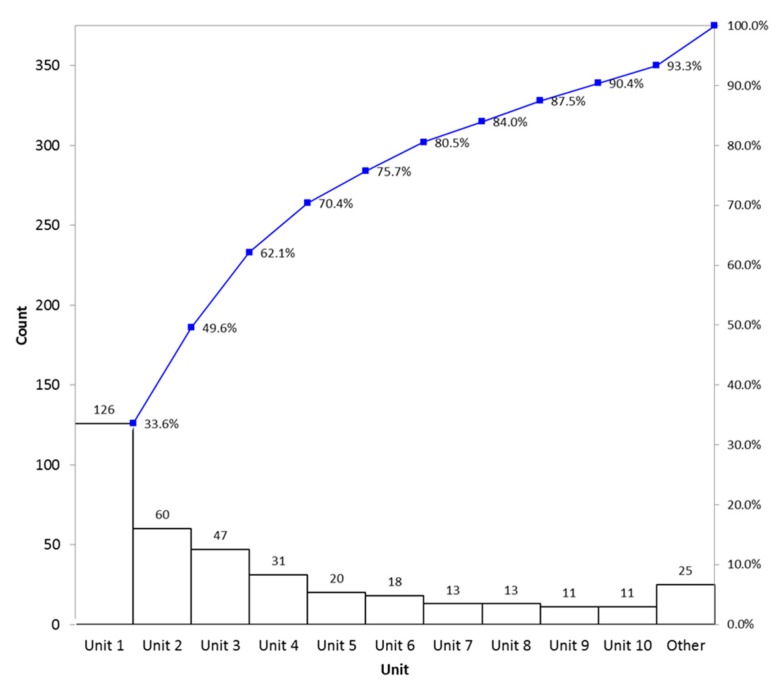
Incomplete tobacco use screens by unit. “Other” contains five individual units.

The data demonstrate wide variability among nursing units in the percent of adolescents screened for tobacco use. Two of the 15 hospital units accounted for approximately 50% of the incomplete tobacco use screening questions, as seen in Figure 4.

## 4. Discussion

### 4.1. Summary and Interpretation of Findings

Our QI project showed that the overall screening for tobacco use among hospitalized adolescents at COA was 90.1% at baseline and remained steady throughout the study. The percentage of adolescents who screened positive for tobacco use was much lower than expected when compared to known Alabama statistics [5]. Adding e-cigarettes as an option to the tobacco use screening questions did increase positive screens for e-cigarette use; however, it did not change the reported percentage of overall tobacco use. There were no patients that reported hookah use. 

With regard to our aim of increasing screening of tobacco use, in our 33 months of data collection, including 21 months of interventions from February 2017 through September 2018, we found that our baseline level of screening was high at 90.1% of adolescent inpatients screened. This higher-than-expected baseline screening was likely due to inclusion of the tobacco use screening questions on the admission Nursing Intake Form, which nurses are mandated to complete within 24 hours of admission. The percent of patients screened for tobacco use fell short of our aim of 95% of hospitalized adolescents screened, and did not increase following educational interventions. The reason for this is likely multifactorial. Based on the nominal group interviews, as well as discussions with nurses from individual units, the etiology is likely related to variability in nursing unit buy-in, multiple competing QI projects, knowledge of the tobacco use screening questionnaire, a workaround allowing completion of the document without asking the questions by clicking “unknown if ever smoked”, and acuity of patient illness making the screen unable to be completed. 

With regard to our second aim of increasing tobacco use reporting to 20%, data collection revealed no change in our baseline vs. intervention period. In our patient population, 4.8% of screened hospitalized inpatients reported use, which was much lower than state and national data [2,3,5,11]. Although the population of adolescent patients admitted to the hospital may have a lower rate of tobacco use, we have no data to suggest tobacco use would be 30% different than the statewide adolescent rate of tobacco use. We postulate that it is most likely associated with issues surrounding privacy and response bias. One finding from our focus group was that the method of asking the tobacco use screening questions varied widely across the institution. Some nurses were asking patients with parents or caregivers in the room, some were asking parents or caregivers, some were printing out the Nursing Intake Form and requesting the parents or caregivers to fill it out while the patient was getting settled into the room, and some were asking the parents or caregivers to step out of the room to ask the patients. The barriers to appropriately asking the questions confidentially prior to the training video were noted in Table 1, in addition to anecdotal evidence that it hindered workflow and time management. Our preferred method, as presented in the training video with a script, is to ask the parent or caregiver to step out of the room to have a confidential conversation with the adolescent patient in order to obtain the most accurate information about their health. 

Finally, though our interventions did not increase the reported percentage of electronic cigarette use to our aim of 10%, we did see an increase in the number of patients reporting e-cigarette use from zero to five patients per month after adding questions specific to e-cigarettes to the tobacco use screen. Even with this increase, there was not an increase in overall reported tobacco use, suggesting that those using e-cigarettes are also using other forms of tobacco (i.e., dual use). This is consistent with published literature that demonstrates that use of e-cigarettes is associated with higher odds of concomitant cigarette use [12]. 

Given the impact of tobacco use, as well as the concerning proportion of high school students that report tobacco and e-cigarette use state-wide and nationally, it is important for providers to take advantage of every opportunity to screen adolescents for tobacco use in order to identify at-risk individuals and provide appropriate tobacco cessation education, including in the hospital setting [4,5]. Counseling and interventions for tobacco use are recommended by the American Academy of Pediatrics and USPSTF [6].

While several studies have evaluated screening in emergency departments and psychiatric wards [13,14,15,16,17,18], very little is known about effective tobacco use screening for inpatient adolescents. One study in Australia found that patients admitted to an adolescent medical unit had the highest proportion of any psychosocial screening completed at 80%, but this did not focus exclusively on tobacco use screening [13]. A study out of Singapore screened admitted adolescents for “at risk” psychological, psychiatric, and medical health issues and reported a 90% response rate [19]. No studies were found that specifically addressed tobacco use screening for adolescent patients in inpatient, non-psychiatric units.

This study has several strengths. First, it encompasses almost 13,000 adolescents admitted to COA over a three-year period. Second, the physicians involved had inter-professional synergy with many of the nursing units, and there was hospital buy-in from the beginning with intramural grant funding, altering of the admission tobacco questions hospital-wide, and mandating the educational video. The initial interventions did have associated cost; however, the last few interventions were changes in process which did not require funding. Third, it incorporated an existing process for all hospitalized patients (i.e., the Nursing Intake Form). Fourth, the data were easily electronically abstracted monthly without bias or subjectivity. 

### 4.2. Limitations

Our work has several limitations. Tobacco use screening relies on accurate self-reporting by the adolescent patient. Teenage patients may not want to reveal their tobacco use due to fear of consequences (e.g., parental or caregiver punishment, judgement from providers), therefore it is likely that our percentage reporting use is falsely low due to response bias. 

The educational video encouraged nursing staff to request confidential interviews with the adolescent for the tobacco use screening, potentially increasing the time it takes for nursing staff to complete the admission process. This is an important balancing measure to consider in future interventions. Additionally, the required tobacco use screening questionnaire had a workaround process in which a nurse could bypass the question without asking the patient. While there was nursing involvement in the study, the main champions of this project were physicians. The physicians engaged nursing in the focus group and ensured that nursing leadership was in support of the project. Finally, nursing units were facing several competing priorities, including other QI projects, which may have affected our project. 

### 4.3. Future Directions

This project is ongoing and has the potential to be impactful. Adolescents who screen positive for tobacco use can be targeted for tobacco use interventions. At out institution, we are already performing these interventions when caregivers report tobacco use. According to USPSTF, any counselling intervention, be it in-person, by phone, in print, or via electronic devices, does reduce the risk for smoking initiation and progression in adolescents [6]. 

We are currently examining workflow in the high-performing nursing units to apply lessons learned to all units in order to improve screening. We are also working with the high-performing units to improve the validity of the tobacco use screen in order to decrease response bias. An option could be to repeat the focus groups in both the high- and low-performing units to see the differing barriers. We are also investigating the potential for the creation of a secure electronic form that the patients could complete on their phone or a hospital tablet, as this has been shown to be the preference in many adolescents [15]. Using a hospital tablet would likely allow for easier Health Insurance Portability and Accountability Act compliance due to difficulties with connecting personal devises to the hospital’s secure network. Another option for improving validity in responses would be to have physicians trained in Home, Education/Employment, Activities, Drugs, Sexuality, Suicide, and Safety (HEADSSS) assessments to obtain this information. Currently, HEADSSS assessments are not done uniformly across all diagnoses in adolescents and the data would have to be manually abstracted through chart review. It is possible, however, that this may be thought of by patients as more confidential and lead to more accurate reporting. While we continue to improve the validity of reported tobacco use, we are additionally considering beginning an intervention for those who report tobacco use with a tobacco-cessation intervention video. These have been shown to be acceptable to parents and adolescents in emergency room literature [20]. Lastly, encouraging patients who screen positive for tobacco use to enroll in the smoking cessation text service “teen.smokefree.gov” could be a useful intervention that would allow continued treatment after discharge [21]. 

## 5. Conclusions

Among a population of hospitalized patients 13 years and older at a tertiary care children’s hospital, overall screening for tobacco use was high at 90.1%, with lower than expected reported tobacco use of 4.8%. The addition of an e-cigarette screening question increased the e-cigarette use reporting, although the overall percent of adolescents reporting tobacco use was unchanged. This study highlights the challenges that exist to valid screening of adolescents for tobacco use in a hospital setting. While education and training are important components of any QI project, ultimately systems-based interventions may be needed to be more effective in process change. 

## Figures and Tables

**Table 1:** Top six themes identified by nursing focus groups as issues related to tobacco use screening completion and accuracy.

Nursing Focus Groups Prioritized Identified Themes
1. Parents and caregivers in the room influences honesty of answers
2. Patient feels judged/judgmental questions
3. Not knowing how to follow up with positive response
4. Not a priority
5. Patient severity/cannot ask
6. Time/too busy

**Table 2:** Definition of data measures obtained from tobacco use screening questions.

Measures	Definition
Tobacco User	Using cigarettes, cigars, pipes, hookah, or e-cigarette
Light Tobacco User	Less than 10 cigarettes or equivalent of cigars/pipes/e-cigs per day
Heavy Tobacco User	More than 10 cigarettes or equivalent of cigars/pipes/e-cigs per day
Percent screened	Number of patients screened divided by number of all adolescents admitted that month
Percent positive	Number of patients reporting tobacco use divided by the total number screened
Reported positive	All patients who were screened and reported: tobacco user, current status unknown, former tobacco user, current every day tobacco user, current some day tobacco user, light tobacco user, heavy tobacco user, current chewing tobacco user
Absolute number of tobacco products used	Total number of patients who reported use of each type of tobacco product
